# Does Re-Partnering Behavior Spread Among Former Spouses?

**DOI:** 10.1007/s10680-021-09589-x

**Published:** 2021-07-09

**Authors:** Zafer Buyukkececi

**Affiliations:** University of Cologne, Albertus-Magnus-Platz, 50923 Cologne, Germany

**Keywords:** Re-partnering, Marriage, Cohabitation, Post-divorce relationships

## Abstract

This study focused on individuals’ re-partnering behavior following a divorce and asked whether divorcees influence each other’s new union formation. By exploiting the System of Social statistical Datasets (SSD) of Statistics Netherlands, I identified divorced dyads and examined interdependencies in their re-partnering behavior. Discrete-time event history models accounting for shared characteristics of divorcees that are likely to influence their divorce and re-partnering behavior simultaneously were estimated. Findings showed that the probability of re-partnering increased within the first two years following a former spouse’s new union formation. Further analyses focusing on formerly cohabiting couples rather than divorcees also revealed significant associations in re-partnering behavior. Following a former romantic partner’s new union formation, women were exposed to risk longer than men, due to men’s quicker re-partnering. These results were robust to the falsification tests. Overall, findings indicate that the consequences of a divorce or breakup are not limited to the incidence itself and former romantic partners remain important in each other’s life courses even after a breakup. With the increasing number of divorcees and changing family structures, it is important to consider former spouses as active network partners that may influence individual life courses.

**Supplementary Information:**

The online version contains supplementary material available at 10.1007/s10680-021-09589-x.

## Introduction

Family formation patterns in Western countries have undergone great changes during the twentieth century (see Buchmann & Kriesi, 2011 for a review). Recent evidence consistently indicates that young adults postpone entry into marriage and parenthood as well as leaving parental home (Billari & Liefbroer, 2010; Gauthier, 2007). Moreover, the prevalence and acceptance of living arrangements such as divorce (Schoen & Canudas-Romo, 2006), remarriage (e.g., Coleman et al., 2000), cohabitation with a new partner following a divorce (e.g., de Graaf & Kalmijn, 2003), and serial cohabitation after a breakup (Eickmeyer & Manning, 2018) have increased noticeably within the last decades. Empirical evidence shows that most divorcees re-partner (Coleman et al., 2000; Elzinga & Liefbroer, 2007; Sweeney, 2002) with a profound preference for cohabitation relative to marriage (Wu & Schimmele, 2005) and across a wider age span (Beaujouan, 2012).

Several studies turn to social diffusion and interaction processes to explain these changes in family life courses (Bongaarts & Watkins, 1996; Hernes, 1972; Kohler et al., 2002; Montgomery & Casterline, 1996; Coale & Watkins, 1986). Kohler and colleagues (2002), for instance, highlight the importance of these two processes in explaining the postponement of parenthood and the emergence of lowest-low fertility in Europe. Accordingly, changes in family formation and living arrangements across time and regions might be driven by social interaction effects that escalate the behavioral impact of socioeconomic changes (i.e., *social multiplier effects*), transitions between equilibriums leading to rapid and persistent changes (i.e., *multiple equilibriums*), and inertia in normative changes (i.e., *status quo enforcement*) such as stepfamily formation.

To elicit these effects at the microlevel, studies have focused on networks such as siblings, friends, and colleagues and examined whether the transition to parenthood (Balbo & Barban, 2014; Buyukkececi et al., 2020; Lyngstad & Prskawetz, 2010), marriage (Buyukkececi & Leopold, 2020), and divorce (de Vuijst et al., 2017) spread among these network partners. Social interaction effects might similarly be relevant in explaining the emergence of new living arrangements such as re-partnering following a divorce or breakup. However, studies on social interaction effects and demographic behavior have been limited to specific transitions (i.e., fertility and divorce) and network domains (i.e., colleagues, friends, and siblings).

The role of former spouses in each other’s later life courses has been overlooked in the literature, although up to 40 percent of marriages in Europe end in divorce (Eurostat, 2016). Most divorcees contact and stay informed about each other even after divorce (Fischer et al., 2005). This might be even more relevant in some contexts such as the Netherlands, a relatively small country with an extensive transport network and low residential mobility levels after separation (Kulu et al., 2020). Moreover, most Dutch divorcees remain in frequent contact (Fischer et al., 2005) and re-partner after divorce (de Graaf & Kalmijn, 2003). Accordingly, former spouses might be influential in newly emerging life-course patterns such as re-partnering. A large body of research has recognized the direct consequences of divorce for various outcomes including demographic behavior (e.g., Wu & Schimmele, 2005), health (e.g., Simon, 2002), risk of poverty (e.g., Smock et al., 1999), and well-being (e.g., Leopold & Kalmijn, 2016). Yet, no studies have investigated post-divorce relationships or social interaction effects in re-partnering.

In this study, I examine the relationship between former spouses’ re-partnering behavior following a divorce in the Netherlands. I use a series of discrete-time event history models and introduce a strategy that accounts for former spouses’ shared characteristics that are likely to influence their divorce decisions and re-partnering simultaneously leading to spurious correlations between divorcees’ re-partnering behavior.

The data come from the System of Social statistical Datasets (SSD), which is an integrated longitudinal database comprising various registers provided by Statistics Netherlands (Bakker et al., 2014). It holds information on the entire Dutch population, including marital and cohabitation histories as well as the timing and duration of each event. Consequently, individuals in the database can be linked to their former spouses through unique individual identifiers. This information is exceptionally suitable to trace divorced dyads’ re-partnering behavior simultaneously and test whether they are related to each other.

## Contact Between Former Spouses

Although marriage is legally ended by a divorce, it does not necessarily mean the end of a social relationship. Contact, as well as attachment between former spouses, may continue in different ways ranging from telephone calls to visits and joint activities (Jacobson, 1983). Using data from a Dutch life-course survey with overrepresented divorced individuals, Fischer and colleagues (2005) reported evidence on post-divorce contact. They found that almost half of the adults surveyed were in contact with their former spouses even after 10 or more years following a divorce.

To gain more insight into the nature and frequency of contact, studies have focused on various determinants (Fischer et al., 2005; Jacobson, 1983; Masheter, 1991) and listed two key factors: duration since the divorce and joint children. Contact between former spouses is influenced by the duration since divorce because divorcees form new economic, emotional, and social ties as time goes by (Jacobson, 1983). Accordingly, attachments between former spouses tend to weaken with the time passed after a divorce, and the intimacy and frequency of relationships diminish over time (Fischer et al., 2005).

Joint attachments of former spouses such as having children also influence post-divorce contact. Former spouses are expected to retain strong social attachments when they have joint children and are likely to remain in frequent contact, especially when their children are still young due to factors such as parental obligations and visiting arrangements (Jacobson, 1983; Masheter, 1997). In contrast, it is easier for former spouses to avoid each other when they do not have children. Fischer and colleagues (2005) show that about 70 percent of former spouses with children were still in contact after 10 years, whereas the proportion of those who maintained contact decreased to 40 percent for divorced couples without children.

Apart from direct contact between former spouses, they may remain informed about each other indirectly through different channels. Mutual acquaintances such as common friends, for instance, might be an important source of information about the life course of a former spouse (Masheter, 1997). Moreover, studies show that new channels for gathering information have emerged with the advent of the internet and social media. College students reported that they used social media to monitor former partners either through direct linkage or indirect connection in social media through common friends (Lyndon et al., 2011). Although no studies have focused on former spouse interaction in that sense, it is plausible that they remain informed about each other through similar channels.

Taken together, considering the high fraction of individuals who stay in direct contact with their former spouses together with the possibility that they indirectly stay informed of each other, divorce can be seen as a dynamic and complex process of family transition where ex-spouses often remain relevant network partners in an individual’s life. Limited evidence, indeed, suggests that preoccupation with a former spouse (Masheter, 1991, 1997) and the status updates of an ex-partner in social media (Lyndon et al., 2011) are associated with individuals’ well-being. Accordingly, most divorcees might be aware of the life-course transitions and behaviors of their former partners, which in turn may influence their own behaviors.

## Former Spouse Influences on Re-partnering

The previous literature has argued that family formation patterns of individuals are not only driven by individuals’ own characteristics and preferences but also influenced by relevant other’s behavior. This literature has predominantly focused on fertility behavior using network domains such as siblings (e.g., Kuziemko, 2006; Lyngstad & Prskawetz, 2010), friends (e.g., Balbo & Barban, 2014), and colleagues (e.g., Buyukkececi et al., 2020; Pink et al., 2014). Studies focusing on other family formation patterns include de Vuijst et al. (2017) and McDermott et al. (2013) where the influence of siblings and peers on divorce behavior are examined, respectively. In another study, Buyukkececi and Leopold (2020) investigated how siblings’ fertility, marital, and divorce behavior are related to each other by not limiting social interactions to the same behavioral domain (e.g., fertility–fertility or divorce–divorce associations). Most of these studies found significant associations between network partners’ family formation behavior.

In this study, I focus on network partners that have not been taken into consideration in the previous literature, namely former spouses. I build on relative deprivation and social comparison theory and qualitative research on social interaction effects and family formation behavior to conceptualize how former spouses may influence re-partnering behavior. Relative deprivation refers to dissatisfaction or resentment as one feels deprived of some desired outcomes compared to standard, real, or imaginary conditions of other people (Crosby, 1976). These comparisons might be with a group or be more specific, local, and interpersonal, which is referred to as personal relative deprivation (PRD).

The comparison references can easily be made in the existence of clear norms as relative deprivation and desired states can be measured by referring to the consensual norms of desirability (Williams, 2017). Yet, if the norms are unclear, vague, or ambiguous the references become less certain. In such a situation, comparisons are made among individuals who are exposed to the same deprivation and share similar attributes as the sociopsychological factors become more important in determining the intergroup references and desired outcomes.

Festinger has proposed a sociopsychological theory of social comparison processes that focuses on how individuals compare their situation with others, especially when they are incapable of evaluating their own situation, opinions, and abilities (Festinger, 1954; Festinger et al., 1950). Individuals assess their own needs and well-being by comparing themselves in important domains with benchmarks provided by the behavior or situation of others. In most cases, individuals are inclined to choose a comparison benchmark that is closer to them for self-evaluation (Bandura, 1994; Wood, 1989), given that more accurate appraisals and diagnostic information are produced when people compare themselves with those who are similar (Festinger, 1954).

Qualitative research, indeed, posits evidence for the contagion of family formation behavior through the social comparison mechanism. Keim and colleagues (2013) showed that individuals report exerted social pressure when fertility was common in the workplace. In a similar perspective, normative parental expectations regarding family formation become more relevant and pressure rises to follow suit when a sibling or a peer fulfills these parental expectations (Bernardi, 2003; Keim et al., 2013).

Re-partnering and the timing of re-partnering are likely to be less certain for divorced individuals in comparison with entry into first union and parenthood. While marriage is related to a matter of “when,” re-partnering is a matter of “if,” especially for individuals who are beyond the normative ages of union formation (de Graaf & Kalmijn, 2003). The loss of well-being and loneliness following a divorce might be reduced by remarrying (Amato, 2000). Moreover, the formation of a new union might be a way to compensate for financial loss as a consequence of divorce (Dewilde & Uunk, 2008). Yet, remarriage may also introduce additional problems such as conflicts between the new spouse and the children (Furstenberg & Cherlin, 1991; Ganong et al., 2019; Hanson et al., 1996).

In the presence of such unclear and ambiguous norms, a former spouse’s behavior may provide a benchmark. The previous literature has shown that individuals consider their partners as relevant comparison references and are influenced by their partners’ life-course outcomes (e.g., Brines & Joyner, 1999; VanYperen & Buunk, 1994). Similarly, spouses also take a step toward forming a family by marrying and usually sharing a deep emotional relationship during the marriage before the divorce (Masheter, 1991). Even though these attachments might weaken or disappear over time, a former spouse’s behavior may also be relevant through social comparison. Individuals may perceive their ex-partners as closer individuals for self-evaluation in terms of life-course transitions, and a formerly married person can be a reference point for re-partnering behavior.

Importantly, there are two ways in which former spouses can be relevant in terms of social comparison. First, relations between divorcees may remain friendly and cordial. Accordingly, former spouses’ life-course transitions and their timing might be influential in a similar way as other network partners such as siblings and colleagues. Following a former spouse’s re-partnering, both own and relevant others’ expectations may rise to follow suit and re-partner. Individuals thereby may change attitudes and behavior toward re-partnering following a former spouse’s union formation.

Second, even in the case of a bad breakup or hostile and preoccupied relations, a former spouse completing the transitions that were initially planned together may increase the pressure on individuals to follow suit and compensate. Individuals may feel deprived and, as a result, a former spouse’s family formation might trigger an individual to form a family as (s)he does not want to be left alone and suffer while the other is striving. Either way, an ex-spouse’s re-partnering might change an individual’s beliefs from “I am not ready yet” to “if (s)he can do it, then I can do it too.”

There might also be gender differences in former spouse effects on re-partnering. Scholars have argued that both men and women traditionally considered that men possessed most of the rights and privileges, and neither the husbands nor wives acknowledged their partners as equals (e.g., Bartley et al., 2005; Fox & Murry, 2000; Scanzoni, 1982). Yet, these perceptions of men and women have significantly altered in the last decades with the women’s movement emphasizing the importance of gender equality. Consequently, men and women started comparing and questioning their position more relative to their counterparts in gender-egalitarian societies.

Gender differences also exist in re-partnering opportunities and behavior due to various factors such as the presence of a child and socioeconomic status. It takes a longer time for women to find a new partner, the likelihood of re-partnering is lower for women than for men at all time intervals (de Graaf & Kalmijn, 2003; Ivanova et al., 2013; Wu & Schimmele, 2005), the difference widens with age, and divorced women may be less attractive as potential partners (Beaujouan, 2012). Arguably, a union dissolution hits women harder than men, and the impact may be even stronger based on prior unions and the duration of these unions (Poortman, 2007). At the same time, women may be more likely to bear the emotional strain of a breakup (Beaujouan, 2012). The marriage market also works in favor of men, who are likely to find new partners over a wider age span (Gelissen, 2004; Goldscheider & Sassler, 2006), and evidence suggests that while the strain of divorce is temporary for men, it is chronic for women (Leopold, 2018). For these reasons, perceptions of unfairness and inequality may differ between men and women following divorce and the re-partnering of a former spouse. A stronger sense of deprivation, in turn, may lead to more motivated attempts to change the situation and restore equality (Greenstein 2009; Smith & Huo, 2014).

Apart from the gender differences in re-partnering, the literature also suggests that the impact of social comparison mechanism on family formation behavior is more relevant to women than men through different channels (Buyukkececi et al., 2020). While men only report the influence of strong ties such as friends and colleagues on family formation behavior, women additionally report the influence of weak ties (Keim et al., 2013). For instance, two German women declared that they were feeling under pressure due to institutional norms and gender roles when their acquaintances who formed a family also expected them to form a family. Consistent with this notion, earlier studies reported women being more eager to social comparison than men in different countries such as the U.S., France, and the Netherlands (Gibbons & Buunk, 1999; Guimond et al., 2007). Taken together, women may experience a stronger sense of deprivation and inequality than men following a former spouse’s re-partnering directly due to the notable gender gap in re-partnering opportunities and behavior. As a result, women may have more motivated attempts to restore equality and re-partner following a former husband’s union formation. Moreover, a former spouse’s re-partnering might be more influential for women directly as evidence indicates that women are more interested in social comparison and indirectly through the strong and weak ties that raise the expectations to follow suit after a former spouse’s re-partnering.

## Other Factors Influencing Re-partnering

Many other factors also affect an individual’s re-partnering behavior and its timing following a divorce. The exiting status from the first union is related to the second union formation and its timing, and divorced individuals have different family formation patterns than cohabiters. Wu & Schimmele (2005) show that divorcees are more likely to remarry than cohabiters with the strong family-oriented traits of marriage and social/economic complexities of divorce, but they have lower overall re-partnering rates: In the short-term (i.e., the first 5 years), divorced individuals are less likely to re-partner, whereas the risk of forming a second union is higher at all time intervals.

Union duration may also have consequences for their re-partnering prospects, though its effects on re-partnering are less clear. On the one hand, it may have a negative effect as individuals are out of the marriage market for a longer time. On the other hand, it may have a positive effect on re-partnering if individuals who are separated from a long union are less willing to live alone. While Bumpass & colleagues (1990) found no effects of former union duration on re-partnering, more recent studies show that longer durations are associated with a higher chance of re-partnering (de Graaf & Kalmijn, 2003; Poortman, 2007; Wu & Schimmele, 2005).

Gender is “the most crucial determinant of the re-partnering process” (Wu & Schimmele, 2005, p. 27). Several studies have found that it takes longer for women to re-partner and their overall likelihood of re-partnering is lower (de Graaf & Kalmijn, 2003; Ivanova et al., 2013; Poortman, 2007; Wu & Schimmele, 2005). Poortman (2007) suggests that these differences are driven by the low benefits and high costs of marriage for women. A body of literature argues men benefit more from marriage, while women do the emotional work in a relationship and be more likely to bear the emotional burden after separation (Beaujouan, 2012; Thompson & Walker, 1989). It might take a longer time to recover from the negative consequences of a union disruption for women, and they may have less desire to form a new relationship. Scholars argue that re-partnering behavior is not limited to preferences, but men have more objections in forming a union with a woman who has been formerly married or has children (Bernhardt & Goldscheider, 2002; South, 2001). Furthermore, opportunities for re-partnering may not be the same for men and women as men are more likely to continue working after cohabitation, marriage, and having children, and workplaces are important contexts for finding a new partner (de Graaf & Kalmijn, 2003; Gelissen, 2004; Goldscheider & Sassler, 2006).

Children add a further dimension to remarriage or re-partnering and may account for differences between men and women. Individuals’ attractiveness to potential partners may decrease in the presence of children because more investment (e.g., potential role as a stepparent) is required in the presence of a child from a previous union (Stewart et al., 2003; Vanassche et al., 2015). Apart from attractiveness, children may influence re-partnering chances in two ways. First, individuals with children will be less interested in forming a new union as their desire to have a child is already met. Second, they will have limited time for leisure activities and fewer opportunities to meet potential new partners due to caring obligations (de Graaf & Kalmijn, 2003; Goldscheider et al., 2009; Koo et al., 1984).

Earlier studies showed that divorced parents are less likely to form a new relationship than childless divorcees, and both the residency and age of children influence re-partnering decisions (Bumpass et al., 1990; Teachman & Heckert, 1985). The consequences of children, however, are not the same for men and women. Most studies have shown that mothers are less likely to form a union than men and childless women, especially in the presence of resident and younger children (Beaujouan, 2012; de Graaf & Kalmijn, 2003; Di Nallo, 2019; Ivanova et al., 2013; Poortman, 2007). Furthermore, Schnor and colleagues (2017) argue that the presence and the impact of children on re-partnering might be underestimated because mothers with sole physical custody might be also more family-oriented and prone to re-partnering and restoring the picture of a complete family. Indeed, their findings show that sole physical custody reduces the probability of re-partnering by 63% when accounting for the selection, whereas this was 33% in conservative estimations that do not consider selection. An exception to these studies which show having children decreases the likelihood of re-partnering is Wu & Schimmele (2005) who found that having young children does not impede women’s second union formation.

The effect of children on men’s re-partnering chances may differ as women are more willing to form a union than men when the potential partner has children because fathers are more reliable partners (Bernhardt & Goldscheider, 2002; Lappegård & Rønsen, 2013; South, 2001). While Stewart and colleagues (2003), and Wu and Schimmele (2005) found that children influence the propensity to re-partnering positively, others (de Graaf & Kalmijn, 2003; Di Nallo 2019; Poortman, 2007) found no effects of children on forming a new union for men. Although findings on the consequences of children for men’s re-partnering are mixed, children play a crucial role in explaining gender differences. As shown by Ivanova and colleagues (2013), gender differences in re-partnering become insignificant when only childless men and women are compared, suggesting that the gender gap in re-partnering is mainly driven by children.

The socioeconomic status of individuals might be relevant in the re-partnering process as union dissolution influences wealth. Marital dissolution lowers household wealth, and the impact is similar in size for men and women, whereas dissolution of cohabiting unions only lowers the household wealth of women (Boertien & Lersch, 2021). Re-partnering might be a way to overcome these negative consequences of a union dissolution for financial distress, and the influence of socioeconomic status might differ by gender (Dewilde & Uunk, 2008). The propensity to remarry low-educated women is no less than their risk of the first marriage, whereas college-educated women have the lowest chances of remarrying (Shafer & James, 2013) as women with low-income compensate for declines in economic well-being by forming a new union (Dewilde & Uunk, 2008). Different from women, the least educated men have the lowest chances of remarrying (Shafer & James, 2013).

## Method

### Data and Sample

I use data from the System of Social statistical Datasets (SSD) of Statistics Netherlands. It is a harmonized longitudinal database consisting of various registers and surveys provided by Statistics Netherlands. The central unit types comprising the database are individuals, households, buildings, and organizations with unique identifiers (Bakker et al., 2014). Through these unique identifiers, datasets can be linked to each other. In this study, I mainly focused on registers. The central database included information on personal identification numbers (PIN, i.e., anonymized citizen service numbers), year and month of birth, gender, and education. This data allowed me to trace both former spouses’ post-divorce re-partnering behavior dynamically and link them to each other.

Information on cohabitation in Dutch registers is available from 1994 onward and based on the municipal population registers and other sources including partner income taxes and social security obtained from various register data sources (van Roon & Harmsen, 2016). Cohabitation relationships are identified by selecting couples who live at the same address and mainly utilizing future information. Accordingly, couples who have been are still or will become married, parents of a common child, or partners for income taxes or social security benefits are identified as cohabiters. Moreover, family members such as siblings are excluded while determining the cohabiters. For people that recently started together in the same address, imputation strategy based on factors such as age difference between the two unattached persons, the combination of sexes, duration of stable address occupation are used to identify whether individuals living in the same household are a cohabiting couple (see van Roon & Harmsen, 2016 for a detailed description). To improve the reliability of the partnership data, December 2016 was chosen as the last month of the observation period in the analyses, but data on cohabitation from 2017 and 2018 were further utilized to identify cohabiting relationships until December 2016.

As SSD holds information on the entire Dutch population, I made a number of selections to create a sample for the analyses. First, I restricted the sample to individuals born between 1970 and 1979. The primary reason for this selection was the extensive set of data available for these cohorts owing to an expansion of the SSD (de Vuijst et al., 2017). Consequently, the age difference between former spouses was set to a maximum of 9 years.

Second, I restricted the sample to divorced individuals. Although union formation patterns have transformed substantially in many Western countries (Eurostat, 2015), unmarried cohabiting individuals were not included in the analyses. This is because cohabiting individuals comprise a highly heterogeneous group ranging from people who have started dating recently to those who have been together for longer periods (Perelli-Harris et al., 2014). Moreover, they may regard cohabitation in different ways ranging from a precursor to marriage to a more favorable way of living than a non-cohabiting relationship (Steele et al., 2005). Marriage, however, is based upon greater commitment and mutual dependence, and higher relationship quality than cohabiting unions (Wu & Schimmele, 2005). Consequently, unmarried cohabiting partners may be less relevant to an individual’s life after separation than former spouses, as a stronger commitment toward each other and traditional meaning attached to marriage might be absent for unmarried partners.

The second reason for only focusing on divorcees was empirical. Given that information on cohabitation is available from 1994 onward in the registers, relationships of cohabitation before this period would not be covered in the analyses. In contrast, only 0.4% of the individuals included in the analyses got divorced before 1994 indicating that almost all coresidential unions following a divorce were captured in the analyses with this strategy. After the identification of divorced individuals, I restricted the study population to individuals who were heterosexually married. Former spouses who remarried to each other (1,586 individuals or 1.1% of the total sample) or had a child together (748 individuals or 0.5% of the total sample) after a divorce were also excluded from the analyses. After these restrictions, the final sample comprised 60,531 dyads (i.e., 121,062 individuals).

In the main analyses, I focused on the re-partnering behavior of divorcees. Yet, it should be noted that cohabitation is notably common in the Netherlands (e.g., Manting, 1994). For that reason, despite the theoretical and empirical research deficits of focusing on cohabiters, I further examined the re-partnering behavior of two groups of cohabiters who are likely to remain relevant for each other following a union dissolution as additional analyses: Cohabiters who (1) lived together at least 3 years and (2) had a child before union dissolution. I focused on first identified romantic partners who are domiciled at the same address that did not marry but experienced a union dissolution and traced their re-partnering behavior. Individuals who had a joint child or started living together again after a breakup were excluded from the analyses.

### Analytical Strategy

The probability of re-partnering after a divorce was estimated by event history analysis based on discrete-time logit models with random effects at the individual level. With the inclusion of random effects, individual variability was defined specifically, and the scope of inference was allowed to be generalized to the entire population (Neter et al., 1996). I also included time-constant and time-varying controls that are likely to influence both re-partnering behavior and the interaction between former spouses.

According to the literature on social interaction effects, similarities in network partners’ behavior might be driven by contextual factors such as shared environment or selection effects apart from the direct influence of a network partner (Manski, 1993). Although including random effects and various controls accounted for these contextual and selection effects to some extent, further considerations were required to disentangle direct former spouse effects.

It has been documented that similar individuals are more likely to get married, and homogamy in marriage occurs along various dimensions including education, ethnicity, age, and even attractiveness (see Kalmijn, 1998, for a comprehensive review). Accordingly, correlations between the re-partnering behavior of former spouses might be due to unrelated but similar life-course trajectories and family formation preferences. For instance, divorcees are more likely to get married than cohabiters because of their family-oriented attitudes (Wu & Schimmele, 2005). The timing of re-partnering might be similar between former spouses due to shared contextual characteristics, family formation preferences, or a common unobserved random shock that are likely to influence their re-partnering behavior simultaneously. If so, similarities in re-partnering are not driven by the direct former spouse effects.

To address this potential source of bias, I considered two additional factors. First, I used robust standard errors clustered at the former couple-level to acknowledge correlations in divorcees’ re-partnering behavior. The inclusion of robust standard errors is based on the assumption “that the errors are uncorrelated across clusters while errors for individuals belonging to the same cluster may be correlated” (Cameron & Miller [2015], p. 320). Second, I considered shared characteristics among former spouses that are likely to affect family formation patterns while examining their re-partnering behavior. To do so, I estimated the probabilities of re-partnering and getting divorced in first marriages—using the whole married Dutch population born in the 1970s—jointly with correlated error terms. Subsequently, the calculated error term in the divorce equation referring to the unobserved shared characteristics of former spouses that were related to the divorce behavior entered as a regressor in the first equation where I estimated the risk of re-partnering (Heckman, 1979). With this strategy, I accounted for unobserved former couple-specific characteristics influencing divorce behavior in the main analyses. The two-step equations for the former spouse effects model took the following form:

1 $$\log \left( {\frac{{r_{i} \left( t \right)}}{{1 - r_{i} \left( t \right)}}} \right) = \alpha T_{i} \left( t \right) + \beta _{1} A_{i} \left( t \right) + \beta ^{\prime}_{2} X_{i} + \beta ^{\prime}_{3} Z_{i} \left( t \right) + \beta _{4} \lambda _{i} + \mathop \sum \limits_{{s = 1}}^{3} \sigma _{s} ^{\prime } C_{s} \left( {t_{i} } \right) + ~\varepsilon _{i}$$

2 $$\Phi ^{{ - 1}} \left( {\Pr \left( {d_{i} = 1} \right)} \right) = \alpha M_{i} + \beta _{1} ^{\prime } X_{i} ^{\prime } + \varepsilon _{i}$$

In Eq. 1, I estimated individual *i*'s risk of re-partnering. The second equation predicted the probability of getting divorced based on similarities between former spouses that are recurrently emphasized in the homogamy literature (see Kalmijn, 1998 for a review). ${r}_{i}$ was the risk of re-partnering $T_{i} \left( t \right)A_{i} \left( t \right)$ were the quadratic functions at time $t$ of individual *i* for duration since the divorce and age in order. Including these functions allowed me to control for both age and duration since the divorce in the models. ${Z}_{i}$ represented time-varying covariates and ${X}_{i}$ was a set of time-constant covariates. ${\lambda }_{i}$ denoted the inverse mills ratio obtained in Eq. 2 to account for shared characteristics of former spouses related to the divorce behavior. ${C}_{s}$ was the main predictor and included three time-varying dummies for the former spouse’s entry into the first coresidential union following a divorce. They took the value 1 if the former spouse entered into cohabitation or marriage in the last 0–11 months, 12–23 months, or 24–35 months and 0 otherwise.

In Eq. 2, I estimated a probit model. ${\Phi }^{-1}$ denoted the inverse of the standard normal cumulative distribution function and $\mathrm{P}\mathrm{r}({d}_{i}=1)$ was the probability of getting divorced until December 2016—i.e., the month of last observation—for individual *i*. It was estimated using a probit model. ${M}_{i}$ was the quadratic function of marital duration. ${X}_{i}^{\text{'}}$ denoted a set of variables related to homogamy and shared by the couples: the absolute value of the age difference between the spouses, whether they have the same educational level, same ethnicity, and same parental marital status. Given that the independent variables entering the model were the same for both former spouses, the calculated error term was also the same for the former spouses allowing me to account for shared characteristics of former spouses that were associated with the divorce behavior. ${\varepsilon }_{ij}$ represented the individual error term.

Together with these models, I further assessed the reliability of my results with a falsification test by matching individuals with unrelated persons based on specific characteristics and examining the correlation in the re-partnering behavior of these matched individuals. This tested whether the associations of former spouses’ re-partnering behavior were (partly) driven by common factors between the former spouses such as the similarities in the timing of life-course transitions (Neugarten, 1979). For instance, while the likelihood of re-partnering is less common in the first five years following a divorce, the probability of forming a second union is higher in all time intervals (Wu & Schimmele, 2005). If former spouses’ interdependencies between re-partnering behavior were driven by such factors, similar relationships should be observed between the matched individuals' re-partnering behaviors.

To equalize the variation of life-course transitions between divorced dyads and unrelated dyads, I performed a conditional assignment. Exact matching was done based on divorcees’ birth composition (i.e., an unrelated partner was born in the same year as the former spouse), year of marriage, and year of divorce. Accordingly, the year of marriage and divorce of the matched individual was the same, and the matched individual’s year of birth was also the same as the former spouse. 108,838 people (i.e., 90% of the original sample) were matched with unrelated individuals with this strategy.

### Measures

The outcome measure was based on the marital and cohabitation registers of the SSD. Like previous studies (e.g., Gałęzewska et al., 2017; Pasteels & Mortelmans, 2017), re-partnering referred to entering a coresidential union. The main reason for focusing on coresidential union was that cohabitation prior to marriage is normative and marriages are often preceded by cohabitation in the Netherlands (Perelli-Harris & Gassen, 2012). Moreover, cohabitation is a broadly used alternative to a marriage similar to other Western countries (Manting, 1994). Focusing on coresidential unions was also favorable empirically for two reasons. First, coresident couple formation and marriage are the viable union forms in the registers. Second, right truncation may occur when distinguishing between marriage and cohabitation, given that cohabiters can marry after the observation period. Yet, as discussed earlier, the meaning attached to remarriage or the impact of a former spouse’s remarriage on an individual might be different from starting to live with a new partner. Accordingly, I also examined how remarriage is associated with a former spouse’s re-partnering behavior as a robustness check, and findings are reported in Appendix.

I created a person-month file and a binary outcome measure for re-partnering. Individuals were defined to be under risk of entering a coresidential union with a new partner after they experienced a divorce. The dummy took the value 1 in the month of the re-partnering event and 0 in all preceding months. As illustrated in Panel a of Table 1, 46% (i.e., 31,078 individuals) of my focus group started cohabiting but did not marry in the observation period. Totally, 67,340 re-partnering events were identified and only 1,820 individuals directly married without prior cohabitation. Entry into cohabitation and marriage, on average, took 32 and 54 months, respectively. Remarried individuals lived with a new partner approximately 26 months prior to marriage. Kaplan–Meier survival curve estimates for transition (1) to re-partnering, (2) to marriage, (3) from living together to marriage, and (4) to re-partnering in the presence and absence of a former spouse’s re-partnering are illustrated in order in Appendix, Panel a-d of Figure A1.

**Table 1:** Descriptive statistics

Panel a: Descriptive information on outcome variables					
*Number of re-partnering events*					
Number of marriages	67,340				
Number of marriages (with no prior cohabitation)	1820				
Average time to re-partnering	32.20				
Average time to marriage in months	53.60				
Average time to marriage in months (cohabiting couples)	25.80				
Share of individuals re-partnered after…	1 year	2 years	3 years	4 years	5 years
	0.16	0.29	0.38	0.44	0.48

Panel b: Overview of the variables (main sample)						Panel c: Overview of the variables (unrelated individuals)				
	Mean	SD	Min.	Max.	Person months	Mean	SD	Min.	Max.	Person months
*Time-varying variables*										
Ever re-partnered	0.01		0	1	5,146,144					
*Ex-spouse re-partnered within…*										
0–11 months	0.10		0	1	5,146,144	0.11		0	1	4,591,998
12–23 months	0.08		0	1	5,146,144	0.08		0	1	4,591,998
24–35 months	0.07		0	1	5,146,144	0.06		0	1	4,591,998
Age	36.14	4.97	15.08	46.92	5,146,144	36.23	4.91	17.58	46.83	4,591,998
Duration since divorce (in months)	42.00	39.87	1	290	5,146,144	41.56	39.42	1	290	4,591,998
Joint child (0–3)	0.08		0	1	5,146,144	0.08		0	1	4,591,998
Joint child (3 +)	0.72		0	1	5,146,144	0.71		0	1	4,591,998
*Number of children*										
0	0.25		0	1	5,146,144	0.25		0	1	4,591,998
1	0.24		0	1	5,146,144	0.25		0	1	4,591,998
2	0.37		0	1	5,146,144	0.38		0	1	4,591,998
3 +	0.13		0	1	5,146,144	0.12		0	1	4,591,998
*Parental marital status*										
Single	0.36		0	1	5,146,144	0.35		0	1	4,591,998
Married	0.46		0	1	5,146,144	0.46		0	1	4,591,998
Previously married	0.19		0	1	5,146,144	0.19		0	1	4,591,998
*Time-constant covariates*										
Female	0.53		0	1	5,146,144	0.53		0	1	4,591,998
High education	0.15		0	1	5,146,144	0.16		0	1	4,591,998
Income (in percentiles)	57.19	24.90	0	100	4,899,514	57.89	24.87	0	100	4,380,192
*Ethnicity*										
Dutch	0.77		0	1	5,146,144	0.79		0	1	4,591,998
Moroccan	0.02		0	1	5,146,144	0.02		0	1	4,591,998
Turkish	0.06		0	1	5,146,144	0.05		0	1	4,591,998
Surinamese	0.04		0	1	5,146,144	0.04		0	1	4,591,998
Dutch Antillean/Aruba	0.01		0	1	5,146,144	0.01		0	1	4,591,998
Other non-Western	0.01		0	1	5,146,144	0.01		0	1	4,591,998
Other Western	0.08		0	1	5,146,144	0.08		0	1	4,591,998
Marital duration	82.50	50.47	0	337	5,146,144	81.56	48.82	0	305	4,591,998
Mother's age at first birth	23.33	4.22	11.67	57.67	4,971,390	23.37	4.19	11.67	55	4,472,840
Parental income (in percentiles)	50.95	26.48	0	99	4,693,810	51.43	26.38	0	99	4,240,892
*Parents' home ownership*										
Own house	0.59		0	1	4,703,853	0.60		0	1	4,250,373
Rent (with allowance)	0.14		0	1	4,703,853	0.13		0	1	4,250,373
Rent (without allowance)	0.26		0	1	4,703,853	0.26		0	1	4,250,373

*Source*: System of Social statistical Datasets (SSD) of Statistics Netherlands

To test for associations between former spouses’ re-partnering behavior, I adapted a similar strategy used in previous studies examining social interaction effects on family formation behavior (e.g., Balbo & Barban, 2014; Buyukkececi et al., 2020). My main predictors were the former spouse’s first entry into cohabitation or marriage with a new partner following a divorce. I created three time-varying dummies. These dummies took the value 1 if the former spouse re-partnered in the past 11 months, 12 to 23 months, and 24 to 35 months, respectively.

I further included a set of time-varying and time-constant controls in the analyses. Time-varying controls included the duration since the divorce, the total number of children, and the presence and age of a joint child as these factors are important determinants of contact between former spouses (Fischer et al., 2005) and re-partnering behavior following a divorce (Poortman, 2007; Wu & Schimmele, 2005). Duration since the divorce was measured by the number of months passed since the divorce.

I created two dummies for the presence of a small child (i.e., whether former spouses had a joint child aged between 0 and 3), and the presence of an older child (i.e., whether former spouses had a joint child older than 3 years) given that contact between former spouses is more frequent (Fischer et al., 2005) and the likelihood of re-partnering is lower (Ivanova et al., 2013) in the presence of a small child. The quadratic function of age was included to account for the time dependency of the processes of marriage and cohabitation. Parental marital status was also considered as it is associated with offspring’s family formation behavior (e.g., Amato, 1996).

Time-constant controls included covariates such as union duration (Wu & Schimmele, 2005), socioeconomic status (Dewilde & Uunk, 2008; Shafer & James, 2013), parental socioeconomic status, and mother’s age at first birth (Fasang & Raab, 2014), which are strongly related to family formation behavior. To control for individuals’ and their parents’ socioeconomic status, I included individuals’ education and their income, and their parents’ income and house ownership, respectively. Income information was in percentiles and available between 2006 and 2010. Accordingly, I took the average income between 2006 and 2010. Gender and ethnicity were also considered as they are relevant determinants of family formation behavior (e.g., Wu & Schimmele, 2005). Panel b and c of Table 1 give an overview of the variables used in the main models and falsification test.

## Results

**Fig. 1:**
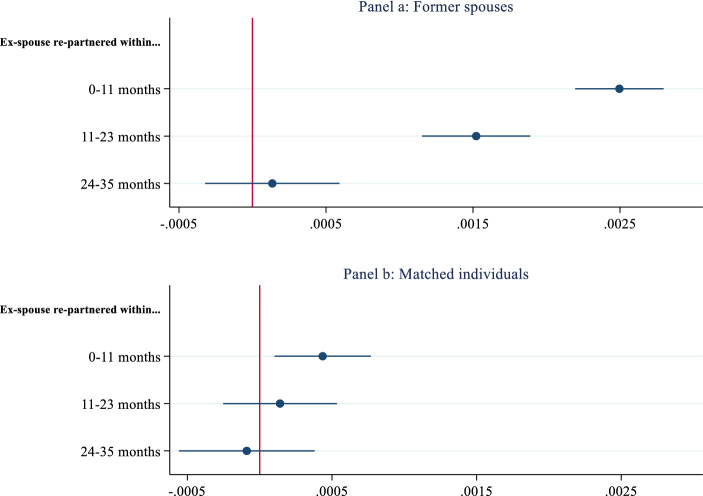
Predicted probabilities of former spouses and matched individuals. Source: System of Social statistical Datasets (SSD) of Statistics Netherlands

Panel a of Fig. 1 presents the estimated main effects for the transition to re-partnering with random effects at the individual-level and controls (estimated coefficients of discrete-time event history model estimates are located in Appendix, Model 1 of Table A1). For the comparability of the models and easier interpretation, discrete changes in predicted probabilities of social interaction dummies are presented in the figures. The model was estimated jointly with the probability of experiencing a divorce to account for the unobserved factors shared by the former spouses that influence divorce and re-partnering behavior simultaneously (the selection equation is located in Appendix, Table A2).

The results showed significant effects of a former spouse’s re-partnering on an individual’s propensity to enter a coresidential union. The effects were significant in the first two consecutive years following a former spouse’s new union formation. They were strongest within the first year and become insignificant after the second year. Transition rates to re-partnering increased by around 0.25 percentage points in a month within the first year following a former spouses’ entry to a coresidential union indicating an individual became (0.25 × 12) % 3 more likely to form a new union in the year after a former spouse re-partnered.

As for the control variables, like previous studies (de Graaf & Kalmijn, 2003; Ivanova et al., 2013; Poortman, 2007; Wu & Schimmele, 2005), notable gender gaps in re-partnering were observed and women were less likely to enter a coresidential union following a divorce than men. I found a curvilinear baseline hazard: The positive association of older age with re-partnering was combined with a small negative effect of age squared suggesting that the weaker the age effects became, the older an individual was. Duration since the divorce had a positive impact on remarriage in the short run but the likelihood of re-partnering decreased in the long run.

Having a child was negatively associated with re-partnering, but having a child younger than 3 increased the likelihood of forming a new union.^1^ In line with the literature (de Graaf & Kalmijn, 2003; Poortman, 2007; Wu & Schimmele, 2005), longer union duration in the former marriage was associated with a higher chance of re-partnering. There were significant ethnic differences in the chances of re-partnering: Dutch individuals more likely to form a new union than other ethnic minorities. Individuals with a Moroccan, Surinamese, and Turkish origin were least likely to form a union after a divorce. The inverse mills ratio was also significant, indicating former spouses’ shared characteristics influencing divorce behavior were also related to the re-partnering behavior. The positive coefficient of the inverse mills ratio signifies that the main models would produce upwardly biased estimates when selection into divorce was not taken into account. This suggests that dissolution-prone individuals were also more likely to re-partner consistent with the previous literature (Lichter & Quian 2008; Lichter et al., 2016).

In Panel b of Fig. 1, I present the falsification test results on matched individuals for the risk of re-partnering. (The complete set of estimates is located in Appendix, Model 2 of Table A1.) This analysis aimed to test that the effects attributed to the behavior of the ex-spouse on family formation behavior did not reflect spurious correlations driven by unobserved shared factors influencing the re-partnering process or normative timing of life-course transitions. The likelihood of re-partnering increased in the first year following the matched individuals’ union formation. Transition rates to re-partnering increased by about 0.05 percentage points suggesting that around one-fifth of the estimated effects in the main models (i.e., 0.25 percentage points) were driven by similarities in normative timing of life-course transitions. Yet, the increase in predicted probability was only significant at a 5% level and around five times weaker than the estimated main effects. These findings supported the notion that the main findings in Panel A of Fig. 1 were not driven by spurious correlations in divorcees’ timing of re-partnering behavior.

**Fig. 2:**
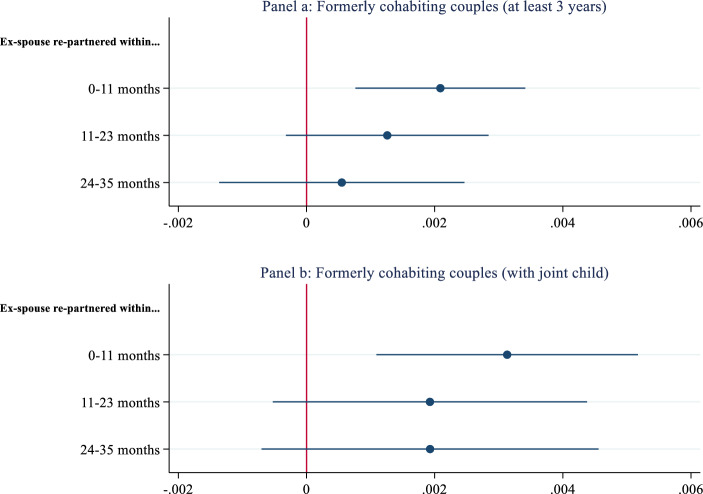
Predicted probabilities of formerly cohabiting couples. Source: System of Social statistical Datasets (SSD) of Statistics Netherlands

Next, I focused on cohabiters who (1) lived at least 3 years together or (2) had a joint child together before the breakup. Estimated main effects are illustrated in Panels a and b of Fig. 2, respectively. (Full estimates are located in Appendix, Models 3 and 4 of Table A1.) Despite the low number of cases in comparison with the main analyses and falsification test, I found significant effects. Individuals were more likely to enter a coresidential union in the first year following a former partner’s union formation. Moreover, the predicted probabilities indicated that the effects were stronger among couples who had a child (i.e., 0.34 percentage points increase) than couples who lived together for at least 3 years (i.e., 0.21 percentage points increase). These increases in predicted probabilities were also about seven and four times larger than the estimated effects using matched individuals.

**Fig. 3:**
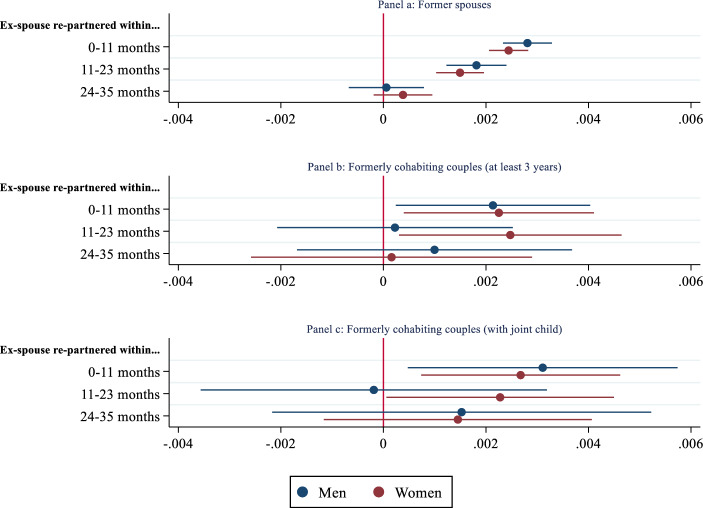
Gender differences. Source: System of Social statistical Datasets (SSD) of Statistics Netherlands

Figure 3 shows how an ex-romantic partner’s new union formation is associated with re-partnering separately for men and women (see Appendix, Table A3 for full estimates). As illustrated in Panel a, no notable differences were estimated among divorced men and women. Moreover, having children decreased the likelihood both for men and women, but the estimated coefficients were stronger for women expectedly. In additional analyses (available upon request), I replicated the models separately by excluding the time-varying number of children dummies. Findings revealed that men’s likelihood of re-partnering increased in the presence of a small child (i.e., 0–3 years old), whereas having an older child decreased the propensity to form a new union. Contrarily, having a child decreased transition rates to re-partnering for women regardless of the child’s age. Yet, the effects were stronger when having a child older than three years.

Gender differences were more noticeable when focusing on previously cohabiting couples. Findings suggested the former romantic partner effects on re-partnering were more long-lasting for women than men. While the estimated effects were only significant within the first year following a former partner’s union formation for men, it was also significant within the second year for women. Apart from the former partner effects, one striking difference between men and women was the role of income in re-partnering. In all three models included in Fig. 3, while men’s likelihood of re-partnering increased with income, it was negatively correlated with forming a new union among women. This is in line with Dewilde & Uunk (2008) who showed that low-income women compensate for decreases in well-being by forming a new union, whereas men with advantageous background characteristics are more likely to re-partner. To strengthen the confidence in my findings, I conducted several robustness checks including distinguishing between marriage and cohabitation, focusing on former spouses who live closer or have a joint child, focusing on remarriage events, excluding “shot-gun” marriages, teenage marriages that ended in divorce, and couples whose marriage lasted less than one year. Findings are reported and discussed in Appendix. Most importantly, models where I utilized remarriage as the outcome variable revealed that former spouse effects on remarriage were more relevant and long-lasting for women than men similar to the models where formerly cohabiting individuals were examined.

## Conclusion

Over the last decades, families in Western societies became more complex through union dissolution, re-partnering/marriage, and stepfamilies (Thomson, 2014). Divorce rates have increased markedly and remained high in Europe during the past half-century (Amato & James, 2010). At the same time, the majority of these divorcees re-partner (Coleman et al., 2000; Sweeney, 2002) and enter into higher-order unions (Elzinga & Liefbroer, 2007). A large body of research emphasizes the importance of social interaction effects in explaining changing family formation patterns (Bongaarts & Watkins, 1996; Hernes, 1972; Kohler et al., 2002; Montgomery & Casterline, 1996; Coale and Watkins, 1986).

Former spouses often remain in touch even after a divorce. Yet, we still know little about post-divorce relationships and the role of former spouses on individuals’ life courses after divorce. An important gap in knowledge concerns re-partnering behavior. Considering the noteworthy direct and indirect contact between former spouses and the growing literature on the relevance of network partners on family formation behavior, former spouses might be important in the emergence of new living arrangements. This might be particularly relevant in the Dutch context, where the likelihood of residential mobility after separation is low (Kulu et al., 2020) and contacts between former spouses are notably common (Fischer et al., 2005). At the same time, about 70% of men and 50% of women re-partner in the first 10 years after divorce (de Graaf & Kalmijn, 2003). Building on this evidence, I investigated whether re-partnering behavior spreads among former spouses in the Netherlands.

Findings showed significant effects and the risk of forming a new union increased in the short-term following a former spouse’s re-partnering suggesting former spouses and their life-course transitions following a divorce remain relevant for individuals. These findings were robust to falsification tests and several robustness checks. Further analyses asserted that not only former spouses but also ex-romantic partners who lived together are notable actors in individuals’ life courses after separation.

Social comparison theory posits that individuals compare themselves with those who are perceived to be similar in the presence of unclear and ambiguous norms and consider their behavior as benchmarks (e.g., Festinger, 1954). Norms and timing of re-partnering might be ambiguous as well for individuals and a former spouse’s “actual” re-partnering behavior may provide a benchmark. Accordingly, divorcees, as well as former romantic partners, may be relevant in the re-partnering processes following a breakup.

No remarkable gender differences in former spouse effects were observed among divorcees. Yet, the estimated effects of previously cohabiting couples were stronger and lasted longer for women than men. Such gender differences were also observed in additional analyses where I examined the remarriage behavior of former spouses. The literature suggests that this is driven by two factors. First, given that perceptions of inequality and greater deprivation trigger attempts to restore equality (Greenstein, 2009; Smith & Huo, 2014), re-partnering of a former spouse might affect women’s feelings of fairness and deprivation more than men’s, due to the large gender gaps in re-partnering that persist in modern societies (e.g., Ivanova et al., 2013; Poortman, 2007). Second, the qualitative literature indicated that women’s family formation behavior is influenced by both strong and weak ties, whereas men are only influenced by the strong ties (Keim et al., 2013). A former spouse’s re-partnering thereby might be more influential on women through more channels.

Above and beyond the former spouse influences on re-partnering, this study provided further empirical evidence on gender differences and the role of children in re-partnering. Overall, women had lower chances of re-partnering than men (de Graaf & Kalmijn, 2003; Ivanova et al., 2013; Poortman, 2007; Wu & Schimmele, 2005). Moreover, like previous studies (Beaujouan, 2012; de Graaf & Kalmijn, 2003; Di Nallo 2019; Ivanova et al., 2013; Poortman, 2007), regardless of the child’s age, women with children had a lower likelihood of re-partnering than childless women. Fathers were also less likely to enter a new union than childless men. In the presence of a small child, however, transition rates to re-partnering increased. This could be driven by the fact that fathers with young children search for a partner who can surrogate the missing mother figure (Bernhardt & Goldscheider, 2002). Yet, it should be noted that custodial arrangements were not considered in the analyses.

I conclude with limitations and suggestions for future research. Despite the benefits of my data for identifying divorcees and determining their re-partnering behavior, I note that I lacked direct information about whether and to what extent former spouses interacted and were informed of each other. However, given the theoretical and empirical background on post-divorce contact and indirect contact (e.g., Fischer et al., 2005), it is plausible that a large majority of the sample were aware of the family formation events of former spouses. Moreover, I note that I was unable to examine the role of relative deprivation and social comparison as the main mechanisms that I expected to give rise to interdependencies among former spouses in the process of re-partnering. As a result, it remains unclear whether and to what extent the interdependencies observed in my analyses were due to social comparison or other factors.

Divorcees are likely to share characteristics such as beauty and income due to the assortative mating, which is likely to affect the chances and timing of re-partnering simultaneously. Although the central findings were supported by further analyses, considerations of attributes shared by the former spouses, and falsification tests in which similar but unrelated individuals were compared, the associations between former spouse’s re-partnering behavior might still be driven by such traits rather than the direct former spouse impact. The main analyses also considered half part of the re-partnering process, given that an individual’s new partner may have a previous union and a former partner who may, in turn, play role in the re-partnering process. Yet, additional robustness checks where I was able to identify new partners who have not been in a union before using the information on the anchor group supported the main findings.

Also, romantic relationships that did not lead to a coresidential union where both partners are registered in the same address were outside the scope of this study with the available registers. Accordingly, new partnerships following divorce might be underestimated, as living apart together (LAT) relationships and relationships where people lived together without being registered to the same address were not considered. Findings focusing on previously cohabiting couples also should be interpreted cautiously. Given that cohabitation data is available after 1994 in the Dutch registers, cohabiting relationships before this period are not captured in the analyses. Furthermore, although the generation of cohabitation data is based on well-built future information and imputation methods, some cohabiting couples might not be identified in the data.

With the increasing availability of register data in various countries (e.g., Denmark, Finland, Norway, and Sweden), it became possible to examine different networks simultaneously, such as siblings, colleagues, and former spouses. If the life-course transitions of these network partners are followed, this will enable researchers to test whether similar associations exist between network partners—including former spouses—in these countries. This, in turn, may shed light on changing family formation patterns across time and regions. Lastly, my analyses emphasize the relevance of former spouses on life-course transitions after a divorce. Other individual outcomes such as health and/or well-being might also be influenced by former spouses’ behavior and post-divorce relationships. It would thereby be worthwhile to study in future research whether different outcomes are also influenced by the behaviors of former spouses.

## Supplementary Information

Below is the link to the electronic supplementary material.

Supplementary file1 (DOCX 92 kb)

## Footnotes

1 Having a child aged between 0 and 3 was negatively correlated with re-partnering and only became positive after including time-varying dummies for the number of children.
