# Supplementary material for: Does Re-Partnering Behavior Spread Among Former Spouses?
Source: Eur J Popul. 2021 Jul 9;37(4-5):799–824. doi: 10.1007/s10680-021-09589-x (PMC8575746; doi:10.1007/s10680-021-09589-x)
Supplement: Supplementary file 1 — Supplementary file1 (DOCX 92 kb) [file 10680_2021_9589_MOESM1_ESM.docx]

**Appendix**

To assess the reliability of my findings, I conducted several robustness checks and present the discrete changes in predicted probabilities of social interaction dummies in the figures (full estimates are available upon request). First, I restricted the analyses to former spouses who are more likely to have contact by examining former spouses (i) who have a common child and (ii) live closer to each other. To identify former spouses living closer to each other, I utilized the distance between former spouses using the geographic coordinates of divorcees’ place of residence. The distance was divided into tertiles and the main analyses were replicated using former spouses who live closer to each other. Predicted probabilities of the main effects obtained from these analyses are presented in the Appendix Panel a and b of Figure A2. While the estimated increases in predicted probabilities of divorcees with a joint child were very similar to the main findings, discrete changes in predicted probabilities of former spouses who live closer to each other were slightly higher. For instance, the transition rate to re-partnering increased by 0.3 percentage points in a month following a former spouses’ union formation.

In the main analyses, I considered individuals’ former spouses’ re-partnering patterns. Yet, an individual’s new partner’s former relationships and partners might also be influential in the re-partnering process. Accordingly, I conducted a robustness check by focusing on individuals whose new partners have not been in a union before (see Panel a of Figure A3 in the Appendix). These findings, however, should be interpreted with caution because full partnership history was only available for the anchor group. Consequently, individuals having new partners outside the anchor group were not included in the analyses, as their new partners’ partnership history was incomplete. With these restrictions, I identified 4,410 individuals and the risk of entering a co-residential union increased in the first year following a former spouse’s re-partnering, despite the low number of cases included in the analysis. In Panel b of Figure A3, I conducted a sensitivity analysis and included six social interaction dummies indicating whether a former spouse formed a new union in the past (i) 0-5, (ii) 6-11, (iii) 12-17, (iv) 18-23, (v) 24-29, and (vi) 30-35 months. Results were similar to the main findings and the effects were significant until the third year following a former spouse’s re-partnering.

In Panel a of Figure A4, I assessed whether a former spouse’s entry into cohabitation and marriage has different consequences on an individual’s re-partnering behavior. The likelihood of re-partnering increased within the first two years following a former spouse’s entry into both cohabitation and remarriage. Yet, the estimated effects of cohabitation were stronger in magnitude. I further replicated the falsification tests using only the most similar unrelated dyads who were matched based on birth composition, year of marriage, year of divorce, and parity and educational composition of the former spouses in Panel b of Figure A4. The social interaction effects of most similar unrelated dyads were insignificant at all time intervals.

Given that ‘shot-gun’ marriages are more likely to end in divorce (Berrington and Diamond 1999) and might imply higher rates of re-partnering (Lichter and Qian 2008) and dissolution-prone individuals who went through short-live marriages are more likely to re-partner (e.g., Lichter and Qian 2008; Lichter et al. 2016), I further conducted four additional robustness checks where the analyses are replicated with the following specifications: (1) without ‘shot-gun’ marriages, (2) without teenage marriages that ended in divorce, (3) without couples whose marriage lasted less than one year, and (4) without all three groups who are more dissolution-prone. Findings were not noticeably altered and are located in the Appendix, Figure A5 and A6.

In Figure A7, I utilized remarriage as the outcome variable and examined whether it is associated with a former spouse’s remarriage and cohabitation. Panel a presents the estimated main effects for the transition to marriage. Results showed significant effects of a former spouse’s marriage on an individual’s propensity to marry at all time intervals. Above and beyond the effects of a former spouse’s marriage, the risk of marriage has increased within the first two years following a former spouse’s entry to cohabitation. Yet, the impact of a former spouse’s cohabitation on the risk of marriage was noticeably weaker than the impact of a former spouse’s marriage.

Panel b of Figure A7 shows how former spouse effects on remarriage and differ by gender. Both men’s and women’s likelihood of marriage increased with a former spouse’s transition to marriage. Yet, the effects on women remained significant in the long-term, whereas men’s risk of marriage was not altered significantly in the third year following a former wife’s marriage. Moreover, the risk of remarriage increased for women within the first two years after a former husband’s entry to cohabitation, whereas a former wife’s cohabitation was significantly associated with remarriage only within the third year suggesting that former spouse effects on remarriage are more relevant for women than men.

In additional robustness checks (not shown), first, I took a randomly selected 10 % of the whole sample in the interests of computation time and convergence of the models and applied a three-level multilevel model with random effects both at the individual and dyadic level. Monthly spells were nested within individuals and individuals were nested within the ex-couple dyads with this specification. Second, given that the social interaction dummies included in the models cover a three-year period, I examined individuals who divorced before December 2013 (i.e., three years before the end of the observation period) and individuals who did not re-partner within the three years following a divorce to test the reliability of the findings. Third, I replicated the falsification tests using former cohabiters rather than divorcees. These individuals were matched with unrelated persons based on former cohabiters’ birth composition, year of entry to cohabitation, and year of union dissolution. Fourth, I controlled for (i) the absolute value of the age difference between the former spouses, (ii) whether they have the same educational level, (iii) same ethnicity, and (iv) same parental marital status to further account for similarities among former spouses. Fifth, rather than using shared characteristics of the former spouses as I did in the main model, I calculated the inverse mills ratio by including individual-specific characteristics. The variables included were the quadratic function of marital duration, age, education, ethnicity, parental income, and parental marital status. Consequently, different from the main models, unobserved individual-specific characteristics related to divorce behavior were considered in these models where I estimated the risk of re-partnering. Lastly, I estimated the risk of re-partnering by focusing on double selectivity (Tunali 1986) in two main underlying decision processes: First, the transition to marriage was estimated using all Dutch individuals born between 1970 and 1979. Second, the transition to divorce was estimated using the married individuals. The findings were not altered qualitatively with these specifications.

**Tables**

Table A1 Discrete-time event history models

|  | Model 1 | | Model 2 | | Model 3 | | Model 4 | |
| --- | --- | --- | --- | --- | --- | --- | --- | --- |
|  | Divorcees | | Unrelated dyads | | Former cohabitors (at least 3 years) | | Former cohabitors (with joint child) | |
|  | Coefficient | Std. Err. | Coefficient | Std. Err. | Coefficient | Std. Err. | Coefficient | Std. Err. |
| Ex-spouse re-partnered within… | | |  |  |  |  |  |  |
| 0-11 months | 0.192 *** | 0.012 | 0.033 * | 0.013 | 0.143 ** | 0.046 | 0.297 ** | 0.093 |
| 12-23 months | 0.117 *** | 0.014 | 0.011 | 0.015 | 0.086 | 0.055 | 0.183 | 0.114 |
| 24-35 months | 0.010 | 0.018 | -0.007 | 0.018 | 0.038 | 0.067 | 0.183 | 0.124 |
| Female | -0.286 *** | 0.010 | -0.278 *** | 0.011 | -0.119 ** | 0.035 | -0.522 *** | 0.114 |
| Age | 0.046 *** | 0.010 | 0.047 *** | 0.011 | 0.058 *** | 0.014 | 0.033 | 0.021 |
| Age squared | -0.002 *** | 0.000 | -0.002 *** | 0.000 | -0.000 *** | 0.000 | -0.000 | 0.000 |
| Duration since divorce (in months) | 0.005 *** | 0.001 | 0.007 *** | 0.001 | -0.000 | 0.002 | 0.017 | 0.009 |
| Duration since divorce squared (in months) | -0.000 *** | 0.000 | -0.000 *** | 0.000 | -0.000 * | 0.000 | -0.000 | 0.000 |
| Joint child (0-3) | 0.107 *** | 0.019 | 0.101 *** | 0.020 | 0.344 ** | 0.123 | 0.394 ** | 0.122 |
| Joint child (3+) | 0.043 | 0.028 | 0.036 | 0.029 | -0.202 | 0.155 | 0.362 * | 0.156 |
| Number of children (ref: no child) | | | |  |  |  |  |  |
| 1 | -0.267 *** | 0.028 | -0.266 *** | 0.029 | -0.111 | 0.150 |  |  |
| 2 | -0.361 *** | 0.032 | -0.364 *** | 0.034 | -0.217 | 0.187 | -0.050 | 0.101 |
| 3+ | -0.346 *** | 0.035 | -0.338 *** | 0.037 | -0.104 | 0.251 | 0.077 | 0.164 |
| *Parental marital status (ref: single)* | | | |  |  |  |  |  |
| Married | 0.013 | 0.010 | 0.015 | 0.011 | 0.003 | 0.040 | -0.089 | 0.103 |
| Previously married | 0.017 | 0.013 | 0.007 | 0.014 | 0.048 | 0.051 | -0.220 | 0.117 |
| High education | 0.052 *** | 0.013 | 0.050 *** | 0.013 | 0.147 *** | 0.042 | 0.133 | 0.145 |
| Income (in percentiles) | 0.002 *** | 0.000 | 0.002 *** | 0.000 | 0.001 | 0.001 | -0.001 | 0.003 |
| *Ethnicity (ref: Dutch)* | | |  |  |  |  |  |  |
| Moroccan | -0.423 *** | 0.031 | -0.423 *** | 0.034 | -0.337 | 0.357 | 0.312 | 0.459 |
| Turkish | -0.565 *** | 0.025 | -0.564 *** | 0.029 | -0.442 | 0.280 | -0.222 | 0.435 |
| Surinamese | -0.465 *** | 0.026 | -0.492 *** | 0.028 | 0.017 | 0.133 | -0.050 | 0.177 |
| Dutch Antillean/Aruba | -0.236 *** | 0.058 | -0.285 ** | 0.063 | -0.607 * | 0.256 | 0.178 | 0.302 |
| Other non-Western | -0.208 *** | 0.041 | -0.225 *** | 0.044 | -0.236 | 0.175 | -0.252 | 0.270 |
| Other Western | -0.143 *** | 0.017 | -0.144 *** | 0.018 | -0.011 | 0.062 | -0.342 | 0.157 |
| Marital duration | 0.001 *** | 0.000 | 0.001 *** | 0.000 |  |  |  |  |
| Mother's age at first birth | -0.006 *** | 0.001 | -0.006 *** | 0.001 | 0.000 | 0.004 | 0.003 | 0.011 |
| Parental income | -0.000 | 0.000 | -0.000 | 0.000 | -0.001 | 0.005 | -0.005 | 0.012 |
| *Parents' home ownership (ref: own house)* | | | | |  |  |  |  |
| Rent (with allowance) | -0.011 | 0.016 | -0.011 | 0.017 | 0.507 | 0.343 | -0.335 | 0.844 |
| Rent (without allowance) | -0.011 | 0.011 | -0.014 | 0.011 | 0.397 | 0.243 | 0.531 | 0.561 |
| Mills ratio | 0.068 ** | 0.021 | 0.079 *** | 0.022 |  |  |  |  |
| rho | 0.052 | 0.007 | 0.053 | 0.007 | 0.000 | 0.001 | 0.294 | 0.119 |
| N | 121,062 | | 108,838 | | 10,304 | | 6,777 | |
| N of spells | 5,146,144 | | 4,591,998 | | 239,960 | | 129,608 | |

Source: System of Social statistical Datasets (SSD) of Statistics Netherlands.

Note: The sample mean was assigned to the missing values of income, mother’s age at first birth, parental income, and dummies for the missing values of these variables – also for parental house ownership – were included. *p<0.05, **p<0.01, ***p<0.001

Table A2 Selection Model for the Probability of Divorce

|  | Probability of Divorce |
| --- | --- |
| Marital duration | -0.006 *** |
|  | (0.000) |
| Marital duration squared | -0.000 *** |
|  | (0.000) |
| Absolute age difference | -0.002 *** |
|  | (0.000) |
| Educational homogamy (Ref: No) | -0.222 *** |
|  | (0.004) |
| Ethnic homogamy (Ref: No) | -0.024 *** |
|  | (0.005) |
| Both partner's parents are not divorced (Ref: No) | -0.188 *** |
|  | (0.004) |
| Constant | 0.197 *** |
|  | (0.010) |
| N | 739,916 |

Source: System of Social statistical Datasets (SSD) of Statistics Netherlands.

*p<0.05, **p<0.01, ***p<0.001

Table A3 Discrete-time event history models (by gender)

|  | Divorcees | | | | Former cohabitors (at least 3 years) | | | | Former cohabitors (with joint child) | | | |
| --- | --- | --- | --- | --- | --- | --- | --- | --- | --- | --- | --- | --- |
|  | Men | | Women | | Men | | Women | | Men | | Women | |
|  | Coefficient | Std. Err. | Coefficient | Std. Err. | Coefficient | Std. Err. | Coefficient | Std. Err. | Coefficient | Std. Err. | Coefficient | Std. Err. |
| Ex-spouse re-partnered within… | | |  |  |  |  |  |  |  |  |  |  |
| 0-11 months | 0.193 *** | 0.017 | 0.212 *** | 0.017 | 0.140 * | 0.063 | 0.162 * | 0.068 | 0.279 * | 0.120 | 0.328 * | 0.120 |
| 12-23 months | 0.124 *** | 0.020 | 0.129 *** | 0.021 | 0.015 | 0.077 | 0.178 * | 0.080 | -0.017 | 0.155 | 0.279 * | 0.138 |
| 24-35 months | 0.004 | 0.026 | 0.033 | 0.025 | 0.065 | 0.090 | 0.011 | 0.101 | 0.139 | 0.169 | 0.178 | 0.163 |
| Age | 0.084 *** | 0.015 | 0.084 *** | 0.014 | 0.058 ** | 0.019 | 0.072 ** | 0.021 | 0.023 | 0.023 | 0.024 | 0.022 |
| Age squared | -0.002 *** | 0.000 | -0.000 *** | 0.000 | -0.000 ** | 0.000 | -0.000 *** | 0.000 | -0.000 | 0.000 | -0.000 | 0.000 |
| Duration since divorce (in months) | 0.004 *** | 0.001 | 0.007 *** | 0.001 | 0.003 | 0.002 | -0.002 | 0.002 | 0.001 | 0.003 | -0.002 | 0.003 |
| Duration since divorce squared (in months) | -0.000 *** | 0.000 | -0.000 *** | 0.000 | -0.000 *** | 0.000 | 0.000 | 0.000 | -0.000 | 0.000 | 0.000 | 0.000 |
| Joint child (0-3) | 0.179 *** | 0.025 | 0.017 | 0.028 | 0.510 ** | 0.166 | 0.200 | 0.186 | 0.580 *** | 0.150 | 0.150 | 0.154 |
| Joint child (3+) | 0.048 | 0.038 | -0.001 | 0.041 | -0.080 | 0.209 | -0.335 | 0.234 | 0.569 ** | 0.190 | 0.077 | 0.191 |
| Number of children (ref: no child) | | | |  |  |  |  |  |  |  |  |  |
| 1 | -0.183 *** | 0.038 | -0.406 *** | 0.041 | -0.064 | 0.202 | -0.235 | 0.225 |  |  |  |  |
| 2 | -0.287 *** | 0.043 | -0.512 *** | 0.047 | -0.153 | 0.251 | -0.395 | 0.283 | -0.082 | 0.103 | -0.158 | 0.107 |
| 3+ | -0.287 *** | 0.047 | -0.498 *** | 0.052 | -0.217 | 0.296 | -0.090 | 0.336 | -0.018 | 0.174 | -0.032 | 0.168 |
| *Parental marital status (ref: single)* | | | |  |  |  |  |  |  |  |  |  |
| Married | 0.013 | 0.014 | 0.008 | 0.016 | 0.002 | 0.054 | -0.010 | 0.060 | 0.046 | 0.097 | -0.172 | 0.105 |
| Previously married | 0.027 | 0.018 | 0.012 | 0.020 | 0.098 | 0.071 | 0.024 | 0.075 | 0.119 | 0.109 | -0.417 *** | 0.112 |
| High education | 0.047 ** | 0.017 | 0.119 *** | 0.019 | 0.253 *** | 0.059 | 0.075 | 0.061 | 0.245 | 0.144 | 0.108 | 0.156 |
| Income (in percentiles) | 0.006 *** | 0.000 | -0.005 *** | 0.000 | 0.009 *** | 0.002 | -0.007 *** | 0.002 | 0.008 ** | 0.003 | -0.015 *** | 0.004 |
| *Ethnicity (ref: Dutch)* | | |  |  |  |  |  |  |  |  |  |  |
| Moroccan | -0.184 *** | 0.040 | -0.646 *** | 0.050 | -0.197 | 0.453 | -0.608 | 0.584 | 0.329 | 0.457 | 0.366 | 0.427 |
| Turkish | -0.261 *** | 0.031 | -0.845 *** | 0.041 | -0.478 | 0.338 | -0.378 | 0.506 | -0.219 | 0.324 | 0.292 | 0.718 |
| Surinamese | -0.299 *** | 0.034 | -0.573 *** | 0.040 | 0.091 | 0.186 | -0.068 | 0.190 | -0.010 | 0.172 | -0.011 | 0.166 |
| Dutch Antillean/Aruba | -0.201 ** | 0.077 | -0.264 ** | 0.090 | -0.447 | 0.295 | -0.819 | 0.518 | 0.071 | 0.250 | -0.010 | 0.305 |
| Other non-Western | -0.176 ** | 0.056 | -0.223 *** | 0.059 | 0.154 | 0.252 | -0.440 | 0.247 | -0.309 | 0.309 | 0.900 | 0.230 |
| Other Western | -0.133 *** | 0.023 | -0.134 *** | 0.025 | 0.018 | 0.083 | -0.032 | 0.093 | -0.197 | 0.142 | -0.243 | 0.146 |
| Marital duration | 0.002 *** | 0.000 | 0.001 | 0.000 |  |  |  |  |  |  |  |  |
| Mother's age at first birth | -0.007 *** | 0.001 | -0.004 * | 0.002 | 0.000 | 0.006 | -0.001 | 0.007 | 0.002 | 0.010 | 0.001 | 0.011 |
| Parental income | -0.001 * | 0.000 | 0.000 | 0.000 | 0.000 | 0.007 | -0.001 | 0.006 | -0.000 | 0.009 | 0.010 | 0.015 |
| *Parents' home ownership (ref: own house)* | | | | |  |  |  |  |  |  |  |  |
| Rent (with allowance) | 0.053 * | 0.020 | -0.077 ** | 0.024 | 0.391 | 0.555 | 0.493 | 0.439 | -0.045 | 0.737 | 0.426 | 0.945 |
| Rent (without allowance) | 0.012 | 0.014 | -0.038 * | 0.016 | 0.411 | 0.358 | 0.296 | 0.329 | 0.300 | 0.476 | 0.916 | 0.649 |
| Mills ratio | 0.018 | 0.028 | 0.127 *** | 0.032 |  |  |  |  |  |  |  |  |
| rho | 0.033 | 0.012 | 0.080 | 0.010 | 0.000 | 0.000 | 0.001 | 0.004 | 0.000 | 0.000 | 0.000 | 0.000 |
| N | 60,531 | | 60,531 | | 5,450 | | 4,854 | | 3,493 | | 3,284 | |
| N of spells | 2,426,393 | | 2,719,751 | | 123,770 | | 116,190 | | 57,968 | | 71,640 | |

Source: System of Social statistical Datasets (SSD) of Statistics Netherlands.

Note: The sample mean was assigned to the missing values of income, mother’s age at first birth, parental income, and dummies for the missing values of these variables – also for parental house ownership – were included. *p<0.05, **p<0.01, ***p<0.001

**Figures**

Figure A1 Survival rates

**** Source: System of Social statistical Datasets (SSD) of Statistics Netherlands.

Figure A2 Predicted probabilities of formal spouses who had a joint child before divorce and who are living closer to each other

 Source: System of Social statistical Datasets (SSD) of Statistics Netherlands.

Figure A3 Predicted probabilities of individuals whose new partner had no prior union formation and sensitivity analysis

 Source: System of Social statistical Datasets (SSD) of Statistics Netherlands.

*Figure A4 Predicted probabilities by distinguishing between marriage and cohabitation of former spouses and most similar matched individuals* Source: System of Social statistical Datasets (SSD) of Statistics Netherlands.

*Figure A5 Predicted probabilities by excluding shotgun and teenage marriages* Source: System of Social statistical Datasets (SSD) of Statistics Netherlands.

*Figure A6 Predicted probabilities by excluding marriages lasted less than one year and all dissolution-prone marriages* Source: System of Social statistical Datasets (SSD) of Statistics Netherlands.

*Figure A7 Predicted probabilities of transition to remarriage*

 Source: System of Social statistical Datasets (SSD) of Statistics Netherlands.
